# Evaluation of [^125^I]α-Bungarotoxin Binding to α7 Nicotinic Acetylcholinergic Receptors in Hippocampus–Subiculum of Postmortem Human Alzheimer’s Disease Brain

**DOI:** 10.3390/receptors4010007

**Published:** 2025-03-20

**Authors:** Allyson Ngo, Fariha Karim, Oshini V. Keerthisinghe, Tram B. Danh, Christopher Liang, Jogeshwar Mukherjee

**Affiliations:** Preclinical Imaging, Department of Radiological Sciences, University of California-Irvine, Irvine, CA 92697, USA

**Keywords:** [^125^I]α-Bungarotoxin, α7 nicotinic acetylcholine receptors, human Aβ plaques, human tau tangles, hippocampus, Alzheimer’s disease, PET imaging

## Abstract

**Background/Objectives:**

Alzheimer’s disease (AD) severely hinders cognitive function in the hippocampus (HP) and subiculum (SUB), impacting the expression of nicotinic acetylcholine receptors (nAChRs) such as the α7-subtype. To investigate α7 nAChRs as a potential PET imaging biomarker, we report the quantitative binding of [^125^I]α-Bungarotoxin ([^125^I]α-Bgtx) for binding to postmortem human AD (n = 29; 13 males, 16 females) HP compared to cognitively normal (CN) (n = 28; 13 male, 15 female) HP.

**Methods:**

For comparisons with common AD biomarkers, adjacent slices were anti-Aβ and anti-Tau immunostained for analysis using QuPath.

**Results:**

The [^125^I]α-Bgtx average SUB/HP ratio was 0.5 among the CN subjects, suggesting higher [^125^I]α-Bgtx binding in the HP gray matter regions. The AD subjects showed overall less binding than the CN subjects, with no statistical significance. A positive correlation was found in the [^125^I]α-Bgtx binding in the AD subjects as the age increased. The Braak stage comparisons of [^125^I]α-Bgtx were made with [^18^F]flotaza binding to Aβ plaques and [^125^I]IPPI binding to Tau. A positive correlation was found between [^125^I]α-Bgtx and [^18^F]flotaza and there was a negative correlation between [^125^I]α-Bgtx and [^125^I]IPPI, implicating intricate relationships between the different AD biomarkers.

**Conclusions:**

[^125^I]α-Bgtx shows complimentary potential as a α7 nAChR imaging agent but needs more preclinical assessments to confirm effectiveness for translational PET studies using α7 nAChR radioligands.

## 1. Introduction

**Figure 1.**
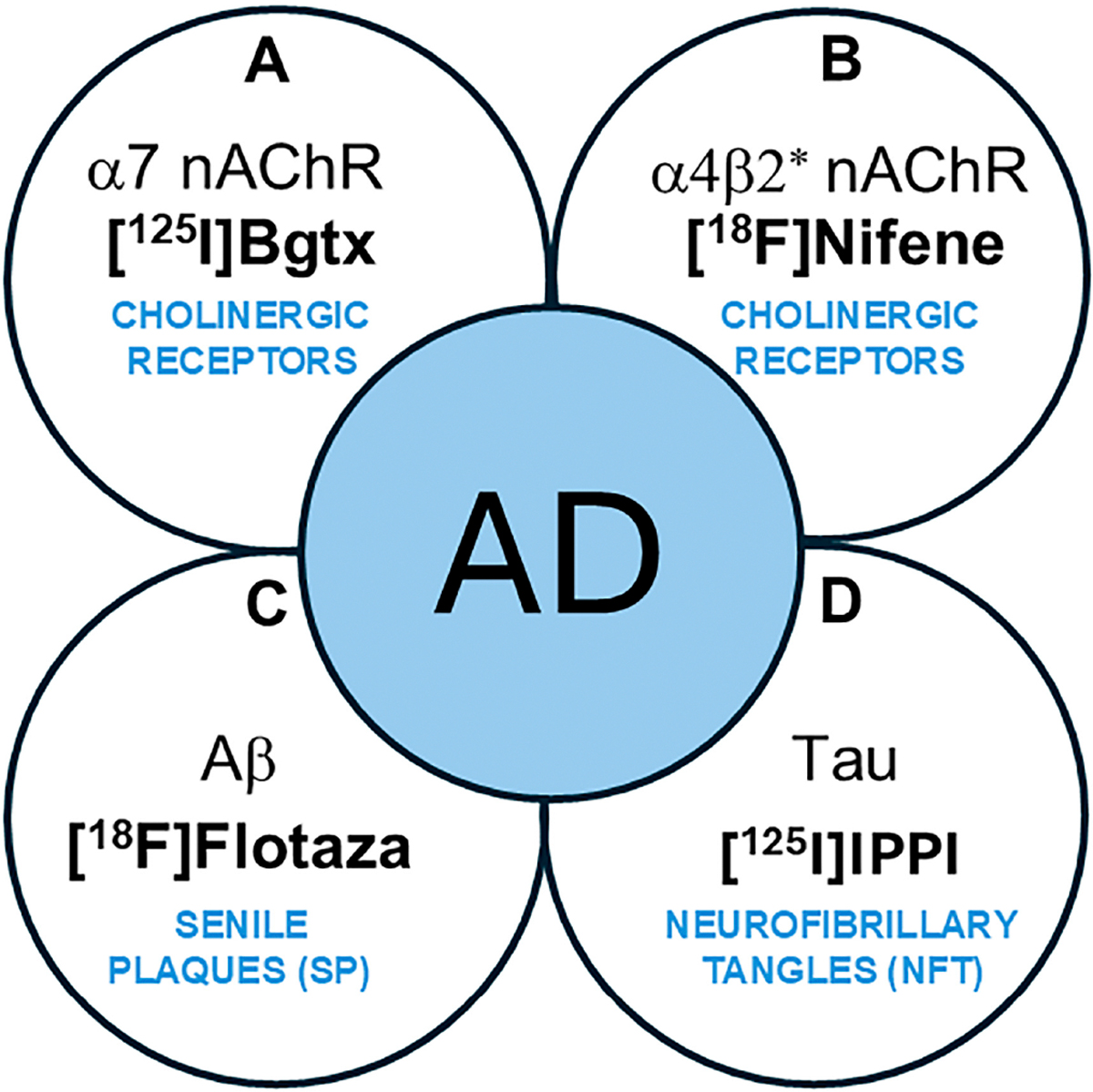
Schematic showing AD biomarkers. (**A**,**B**). Cholinergic abnormalities are being investigated as potential biomarkers. (**A**). α7 nAChRs using [^125^I]α-Bgtx have been evaluated in this work. (**B**). α4β2* nAChRs using [^18^F]nifene and other radiotracers have been reported; (**C**). PET imaging of Aβ plaques (senile plaques, SPs) is currently used in AD subjects. [^18^F]Flotaza was used in this work as an Aβ plaque imaging agent. (**D**). PET imaging of Tau (neurofibrillary tangles, NFTs) is currently used in AD subjects. [^125^I]IPPI was used in this work to evaluate Tau.

Nicotinic acetylcholine α7-subtype receptors (α7 nAChRs) are pentameric ligand-gated ion channels implicated in Alzheimer’s disease (AD) pathology [1,2]. Located in presynaptic and postsynaptic neurons of brain regions including the cerebral cortex and hippocampus (HP) [3], α7 nAChRs are important mediators of intracellular signaling and neurotransmitter release, which are necessary for maintaining normal cognitive function [4,5]. In addition, studies have identified the receptor’s contribution to neuroprotection, synaptic plasticity, and other factors affecting neuronal survival and typical immune responses [6]. A role of α7 nAChRs in neurodegeneration has been suggested in which α7 nAChRs in AD may be influenced by the accumulation of amyloid plaques (Aβ) and neurofibrillary tangles (NFTs) containing Tau protein (Figure 1) [7,8]. As such, Aβ plaques and NFTs are both common AD biomarkers currently necessary for clinical definition, staging, and indications of therapeutic interventions [9,10]. In AD progression, oligomeric Aβ accumulates and may directly interact with α7 nAChRs [11,12]. Neurochemical abnormalities and potential changes in α7 nAChRs along with other cholinergic pathways in AD may be associated with cognitive impairment and emphasize the importance of further investigating the receptor’s role in AD.

To better visualize α7 nAChRs, the involvement of positron emission tomography (PET) imaging and the development of radioligands are being investigated. [^18^F]ASEM was one of the first PET radioligands to successfully demonstrate the ability to bind with high specificity to α7 nAChRs in human subjects [13]. Previous research has identified [^18^F]ASEM binding in mild cognitive impairment (MCI), suggesting [^18^F]ASEM as a promising radioligand with the potential to draw relationships between α7 nAChR availability and MCI [14]. PET imaging of α7 nAChRs with radioligands may assist in the effective detection of neurochemical abnormalities and diagnostic assessments of AD. Although a significant amount of knowledge has been garnered on α7 nAChRs, PET studies on AD have not been sufficiently reported.

Extensively used to measure the α7 nAChR concentration in the brain, α-bungarotoxin (α-Bgtx) is a familiar antagonist composed of a 74-amino-acid toxin extracted from the venom of Bungarus multicinctus snakes [15]. α-Bgtx has been considerably researched and its mechanism of action is well understood, including its high affinity for α7 nAChRs in the mammalian CNS, making it worth investigating as a possible radioligand for measuring the receptor’s concentration in neurological diseases [16,17]. Specifically, the previous use of radioligand [^125^I]α-Bgtx has been successful in detecting the reduced α7 nAChR concentration in the brain of schizophrenic patients [18]. Using [^18^F]ASEM, decreased hippocampal α7 receptor availability has been reported in recent-onset psychosis [19]. Despite the volume of experiments where α-Bgtx is used, little research has been performed on α-Bgtx’s ability to act as an imaging agent for α7 nAChR in postmortem AD brains. This study was undertaken to measure the in vitro binding of [^125^I]α-Bgtx to α7 nAChR sites in AD subjects compared to cognitively normal (CN) subjects and assess potential relationships between other AD biomarkers such as Aβ plaques and Tau tangles using [^18^F]flotaza and [^125^I]IPPI, respectively (Figure 1) [20]. The findings of this study on α7 nAChRs using [^125^I]α-Bgtx may have implications on future PET studies because of the similarities in the binding of [^125^I]α-Bgtx with ASEM analogs [21].

## 2. Materials and Methods

### 2.1. General Methods

[^125^I]α-bungarotoxin was purchased from American Radiolabeled Chemicals, Inc., St Louis, MO, USA, for autoradiographic studies, in which tissue samples were exposed on storage phosphor screens. Cyclone phosphor autoradiographic imaging system (Packard Instruments Co., Boonton, NJ, USA) and Optiquant Imaging System software (Version 4.00.01) were used for analysis.

### 2.2. Postmortem Human Brain

Human postmortem brain tissue sections of HP plus SUB (known to contain abundant NFTs and Aβ plaques, [22]), which were 10 μm thick, on Fisher slides, were obtained from Banner Sun Health Research Institute (BHRI), Sun City, AZ, USA, from the brain tissue repository for in vitro experiments [23]. Age- and gender-matched AD brain and CN brain tissue samples were used for the study. A total of 28 CN (13 males (age 71–97) and 15 females (age 53–95)) and 29 AD (13 males (age 70–91) and 16 female (age 59–93)) subjects were used in this study (Table 1). Brain sections were stored at −80 °C. All postmortem human brain studies were approved by Institutional Biosafety Committee of University of California, Irvine.

### 2.3. Immunohistochemistry

Immunostaining for Aβ and Tau of all brain sections was carried out by University of California, Irvine, Pathology services using Ventana BenchMark Ultra protocols. All IHC stained slides were scanned using the Ventana Roche instrumentation (Ventana Medical Systems, Oro Valley, AZ, USA) and analyzed using QuPath (version QuPath-0.4.2) [20,22].

### 2.4. Radiopharmaceuticals

[^125^I]α-Bgtx: [^125^I]α-Bgtx was purchased from American Radiolabeled Chemicals, Inc. Radiochemical purity was >98% and chemical purity was found to be >95% with a measured molar activity > 70 GBq/μmol. [^18^F]Flotaza: [^18^F]Flotaza (measured molar activity > 70 GBq/μmol) is a radiotracer for imaging Aβ plaques and exhibited selective binding to human AD brain Aβ plaques [22]. [^125^I]IPPI: [^125^I]IPPI (molar activity > 500 GBq/μmol) is a radiotracer for imaging human AD Tau [20].

### 2.5. [^125^I]α-Bgtx Autoradiography

Autoradiography of [^125^I]α-Bgtx in mice and rat brain slices was previously reported [25]. The horizontal brain slices were preincubated in 50 mM Tris HCl, pH 7.3, containing 0.1% bovine serum albumin (BSA) at room temperature for 30 min. After the preincubation, the slices were incubated with [^125^I]α-Bgtx (0.2 nM, molar activity > 70 GBq/μmol) at room temperature for 120 min. Nonspecific binding was measured in separate chambers in the presence of 300 μM nicotine. After incubation, slices were washed three times (10 min each wash) with ice-cold Tris buffer, pH 7.3, followed by a quick rinse in cold (0–5 °C) deionized water. The brain sections were air-dried, exposed for a week on a phosphor film, and then placed in the Phosphor Autoradiographic Imaging System/Cyclone Storage Phosphor System (Packard Instruments Co.). Regions of interest (ROIs) were drawn on the slices and the extent of binding of [^125^I]α-Bgtx was measured in DLU/mm^2^ values provided by the OptiQuant acquisition and analysis program (Packard Instruments Co.).

### 2.6. Image Analysis

All ROIs in the GM and WM autoradiographic images of [^125^I]α-Bgtx were quantified by OptiQuant in DLU/mm^2^. Immunostained sections were analyzed using QuPath. GM and WM binding of [^125^I]α-Bgtx in AD and CN subjects were measured.

### 2.7. Statistical Analysis

Group differences between AD and CN subjects were assessed using average GM/WM ratios and were determined using Microsoft Excel 16 and GraphPad Prism 9. Statistical power was determined with Student’s *t*-test and *p* values of <0.05 were considered to indicate statistical significance. Spearman’s correlation was carried out to assess aging effects.

## 3. Results

### 3.1. [^125^I]Bgtx Binding in Hippocampal Versus Subiculum Regions

**Figure 2.**
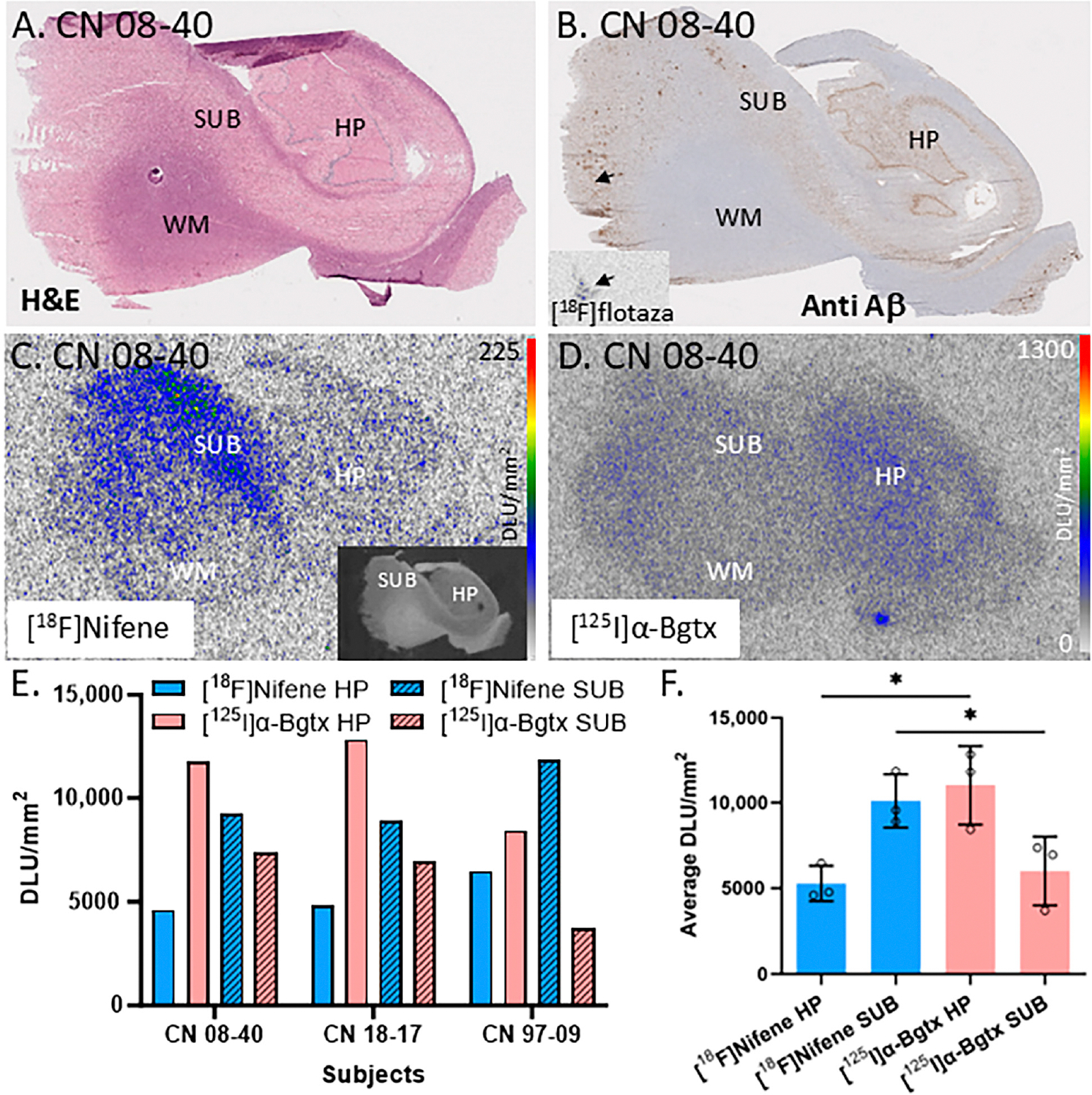
Comparisons between α7 nAChRs and α4β2* nAChRs in the hippocampus–subiculum (HP-SUB): in vitro human HP-SUB brain slices of CN subjects with [^125^I]α-Bgtx and [^18^F]nifene binding. (**A**). H&E-stained CN 08–40 brain section indicating HP, SUB, and white matter (WM) regions. (**B**). Anti-Aβ immunohistochemical staining of CN 08–40 measuring Aβ plaque with trace amount (arrow), confirmed by [^18^F]flotaza autoradiography (inset). (**C**). Autoradiogram of [^18^F]nifene binding in adjacent brain section of CN 08–40 shown in inset (scale bar 0–225 digital light units per square millimeter (DLU/mm^2^)). (**D**). Autoradiograph of adjacent brain section of CN 08–40 showing HP and SUB regions used for [^125^I]α-Bgtx binding (scale bar 0–1300 DLU/mm^2^). (**E**). [^18^F]Nifene and [^125^I]α-Bgtx binding to HP and SUB regions in 3 subjects: CN 08–40 (male), CN 18–17 (female), and CN 97–09 (female). (**F**). Averaged [^18^F]nifene and [^125^I]α-Bgtx binding in DLU/mm^2^ to HP and SUB regions of same 3 subjects (individually represented by circles). Unpaired two-tail parametric *t*-tests calculated statistical significance (*) between [^18^F]nifene and [^125^I]α-Bgtx binding to HP (*p* value = 0.0172) and SUB (*p* value = 0.0491).

Figure 2A is an H&E-stained section of a male CN subject’s brain with labels for the hippocampal (HP), subiculum (SUB), and white matter (WM) regions. The slice from subject CN 08–40 (autopsied subjects listed by year and number, e.g., 08–40 is the 40th autopsy performed in 2008) was labeled by [^18^F]flotaza showing low levels of Aβ plaques in the gray matter (GM; Figure 2B), consistent with [^18^F]flotaza autoradiography (Figure 2B inset), while the WM regions showed near-background levels. The autoradiogram of [^18^F]nifene displays binding to α4β2* nAChRs (Figure 2C), while the autoradiogram of [^125^I]α-Bgtx displays binding to α7 nAChRs (Figure 2D). The regions of interest for the SUB and HP areas were drawn for three subjects to compare the amount of [^18^F]nifene and [^125^I]α-Bgtx binding (Figure 2E). The average SUB/HP ratio for [^18^F]nifene binding was 1.9, suggesting higher binding of [^18^F]nifene to α4β2 nAChRs in the SUB regions compared to that in the HP. On the other hand, the SUB/HP ratio for [^125^I]α-Bgtx binding to α7 nAChRs was 0.5, suggesting higher levels of α7 nAChRs in the HP, confirmed by [^125^I]α-Bgtx binding. These differences are visually evident in Figure 2C,D. A significant difference was found between the [^18^F]nifene (α4β2 nAChR) and [^125^I]α-Bgtx (α7 nAChR) average binding to the HP (*p* = 0.017) and SUB regions (*p* = 0.049) (Figure 2F).

These human brain findings on regional differences in the two receptor subtypes, [^18^F]nifene (α4β2 nAChR) and [^125^I]α-Bgtx (α7 nAChR), are consistent 3/20/2025with our recently reported results in mice and rat brain slices on the differences in [^125^I]α-Bgtx and [^18^F]nifene between the HP and SUB [25]. Thus, there appeared to be similarities in the distribution in the HP and SUB of these two brain nAChR subtypes across species.

### 3.2. CN Female and CN Male Human Postmortem Subjects

**Figure 3.**
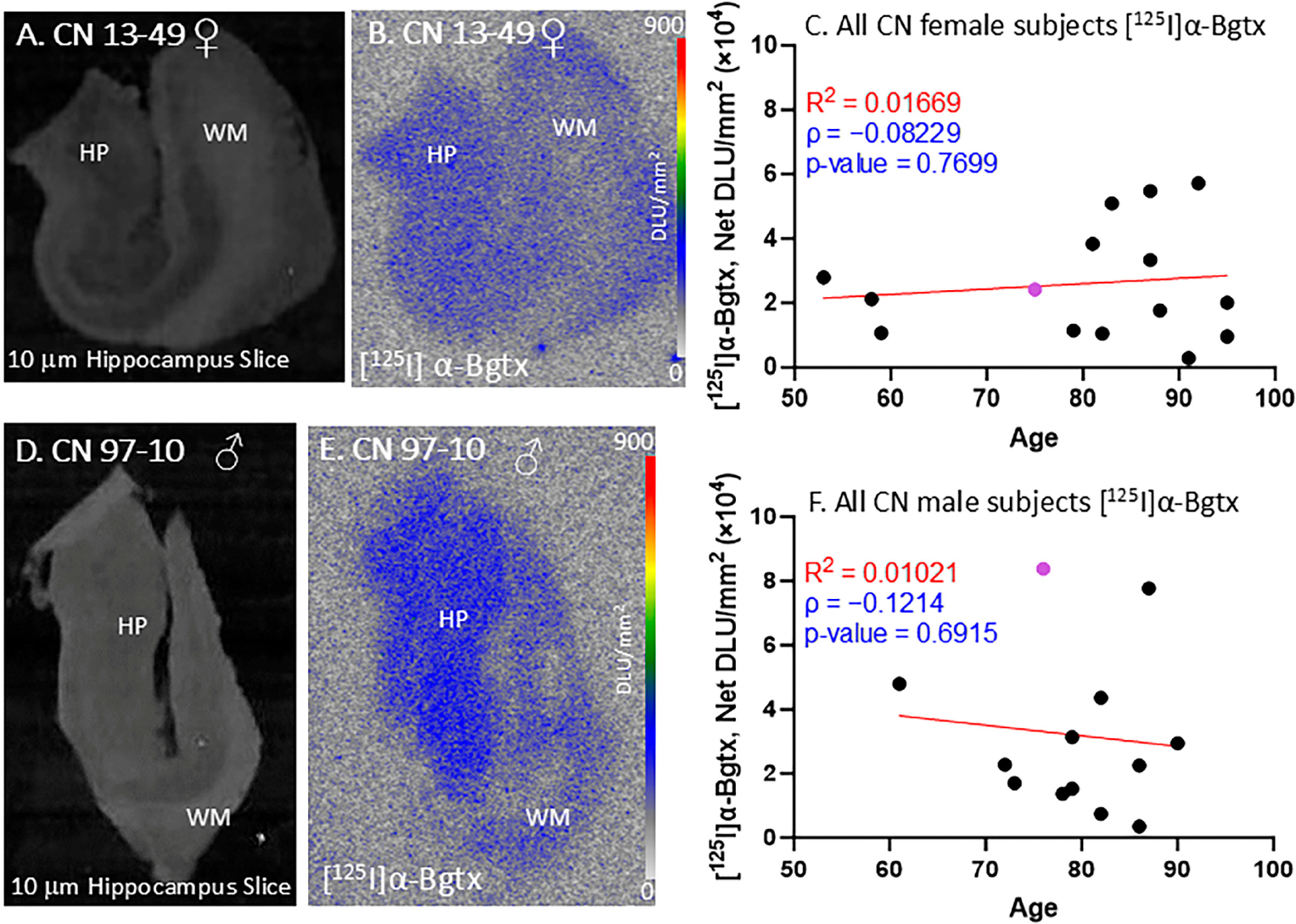
[^125^I]α-Bgtx in CN subjects: in vitro human HP brain slices of representative female and male subjects. (**A**). The HP slice scan of CN female subject 13–49 mapping the distribution of gray matter (GM) and white matter (WM). (**B**). Autoradiograph of [^125^I]α-Bgtx showing higher binding to GM regions of SUB and HP. [^125^I]α-Bgtx autoradiography scale bar: 0–900 DLU/mm^2^. (**C**). [^125^I]α-Bgtx binding correlated with age of all CN female subjects, n = 15 (Spearman’s ρ= −0.08229; *p* value = 0.7699). The purple-colored point indicates CN 13–49. (**D**). The HP slice scan of CN male subject 08–40 mapping the distribution of GM and WM. (**E**). Autoradiograph of [^125^I]α-Bgtx, showing higher binding to GM regions of SUB and HP. [^125^I]α-Bgtx autoradiography scale bar: 0–900 DLU/mm^2^. (**F**). [^125^I]α-Bgtx binding correlated with age of all CN male subjects, n = 13 (Spearman’s ρ = −0.1214; *p* value = 0.6915). The purple-colored point indicates CN 97–10.

All of the female and male CN subjects were observed for [^125^I]α-Bgtx binding to α7 nAChRs (Figure 3). The CN subjects were largely found to be free of Aβ plaques, measured using [^18^F]flotaza [22], and free of NFT Tau, measured using [^125^I]IPPI [20]. All of the female and male CN subjects displayed extensive amounts of α7 nAChRs in the GM regions. [^125^I]α-Bgtx consistently bound to α7 nAChRs in the GM regions, as visually indicated by the autoradiograph of subjects CN 13–49 (Figure 3A,B) and 97–10 (Figure 3D,E). The ages of both the female and male subjects in this study spanned over at least three decades. All of the female (n = 15; Figure 3C) and male (n = 13; Figure 3F) CN subjects exhibited similar levels of [^125^I]α-Bgtx dispersion in binding in the GM hippocampal regions. Because of this dispersion in binding, the aging effects were not clear. Recent findings with [^18^F]ASEM PET have suggested a higher availability of α7 receptors in older CN individuals [26].

### 3.3. AD Female and AD Male Human Postmortem Subjects

**Figure 4.**
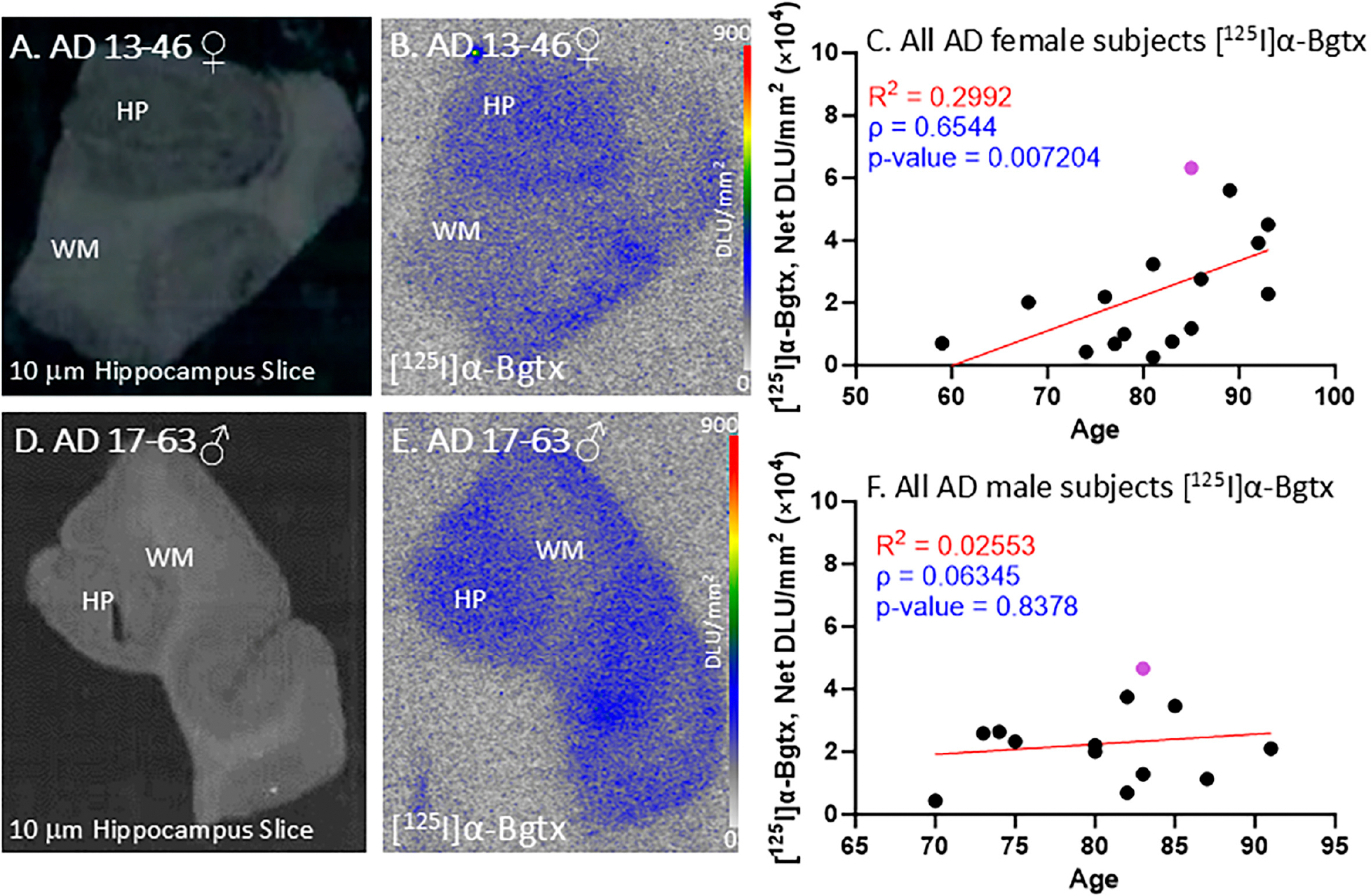
[^125^I]α-Bgtx in AD subjects: in vitro human HP brain slices of representative female and male subjects. (**A**). The HP slice scan of AD female subject 13–46 mapping the distribution of GM and WM. (**B**). Autoradiography of [^125^I]α-Bgtx showing binding to GM regions in SUB and HP. (**C**). [^125^I]α-Bgtx binding correlated with age of all AD female subjects, n = 16 (Spearman’s ρ = 0.6544; *p* value = 0.007204). The purple-colored point indicates AD 13–46. (**D**). The HP slice scan of AD male subject 17–63 mapping the distribution of GM and WM. (**E**). Autoradiography of [^125^I]α-Bgtx showing binding to GM regions in SUB and HP. (**F**). [^125^I]α-Bgtx binding correlated with age of all AD male subjects, n = 13 (Spearman’s ρ = 0.06345; *p* value = 0.8378). The purple-colored point indicates AD 17–63.

All of the female AD subjects were positively immunostained and confirmed with autoradiographs to be positive for Aβ plaques and Tau. Figure 4A–C demonstrate [^125^I]α-Bgtx binding in female subject AD 13–46 (Figure 4B) with a brain slice scan indicating the GM and WM regions (Figure 4A). The [^125^I]α-Bgtx binding levels in the AD female subjects were consistently higher in the GM than WM regions. Specifically, the HP region visually reveals high [^125^I]α-Bgtx binding in the brain slices (Figure 4B). As the AD female subjects aged, there was an overall increase in the [^125^I]α-Bgtx binding over three decades (Figure 4C).

All of the male AD subjects were also confirmed with autoradiographs to be positive for Aβ plaques and Tau. Figure 4E displays the [^125^I]α-Bgtx binding in male subject AD 17–63 with an autoradiograph of the representative brain slice scan with labels for the GM and WM regions (Figure 4D). In all of the AD male subjects, there were consistently higher [^125^I]α-Bgtx binding levels in the GM than those in the WM regions. With increasing age, the AD male subjects experienced a small increase in [^125^I]α-Bgtx binding over two decades (Figure 4F). Thus, in both the female and male AD subjects, a trend towards the increased availability of α7 receptors was observed.

### 3.4. CN-AD Gender Comparisons of [^125^I]α-Bgtx

**Figure 5.**
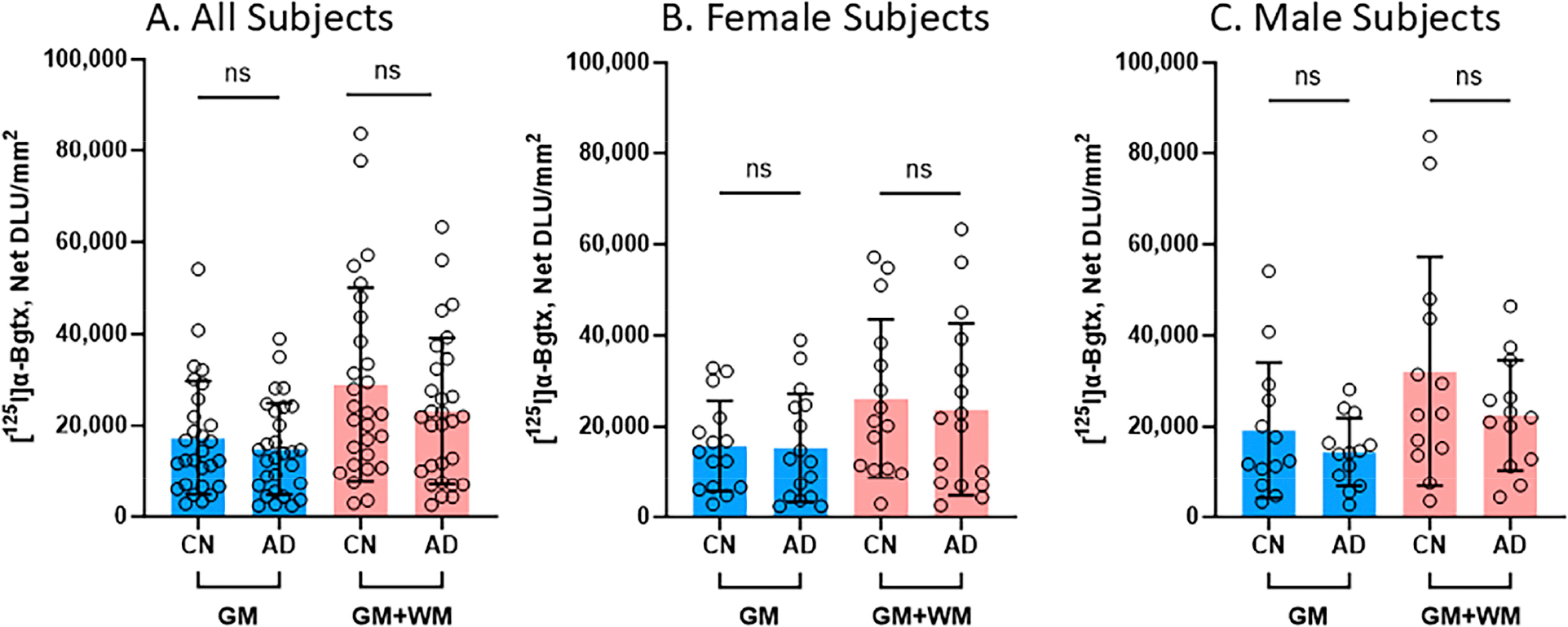
[^125^I]α-Bgtx binding comparisons between CN and AD subjects: Average [^125^I]α-Bgtx total binding (GM) and binding in the entire slice (GM + WM). Each parameter tested statistical significance between CN and AD subjects with unpaired two-tail parametric *t*-tests. ns = not significant. The circles represent individual subjects. (**A**). All subjects (female and male) for GM and GM + WM. (**B**). Female subjects for GM and GM + WM. (**C**). Male subjects for GM and GM + WM.

Several comparisons were made between the CN and AD subjects in regard to [^125^I]α-Bgtx binding in the GM and the entire slice (GM + WM), as shown in Figure 5. In all of the subjects, regardless of gender, there were no significant differences between the CN and AD subjects in the binding in the GM and GM + WM (Figure 5A). Despite there being no significance, the CN subjects consistently displayed higher binding than the AD subjects. A reduction of 14% in [^125^I]α-Bgtx binding in the GM of the AD subjects was observed, while the entire slice exhibited a 19.8% decrease in [^125^I]α-Bgtx binding in the AD subjects (Figure 5A). When comparing the female subjects, there was a decrease of 2.4% in [^125^I]α-Bgtx binding in the GM of the female AD subjects while the GM + WM showed a reduction of 9.2% (Figure 5B). There was a 25.1% decrease in the male AD subjects in comparison to the male CN subjects for [^125^I]α-Bgtx binding in the GM (Figure 5C). The male subjects experienced the greatest difference for [^125^I]α-Bgtx binding in the GM + WM, with a 29.9% decrease in the AD subjects compared to that in the CN subjects.

### 3.5. Braak Stage Comparisons of [^125^I]α-Bgtx to [^18^F]Flotaza and [^125^I]IPPI

**Figure 6.**
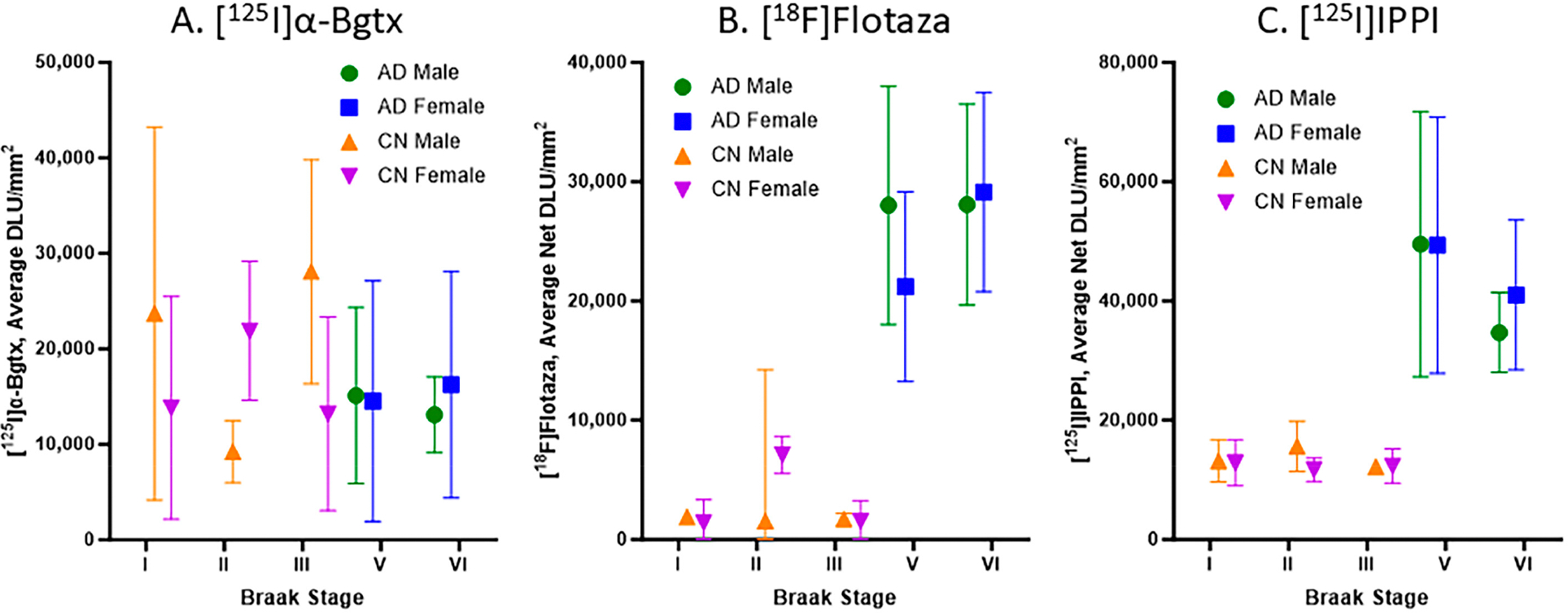
Braak stage plots of [^125^I]α-Bgtx, [^18^F]flotaza, and [^125^I]IPPI binding in human HP: (**A**). [^125^I]α-Bgtx average binding to α7 nAChRs in CN and AD subjects with respect to Braak stages. (**B**). [^18^F]Flotaza average binding to Aβ plaques in CN and AD subjects with respect to Braak stages. (**C**). [^125^I]IPPI average binding to Tau in CN and AD subjects with respect to Braak stages.

Braak stages I, II, III, V, and VI were assigned to all of the CN and AD subjects in this study, with no subjects in Braak stage IV. Figure 6 consists of plots for the relationship between the Braak stage and the binding of [^125^I]α-Bgtx, [^18^F]flotaza, and [^125^I]IPPI in all of the subjects. Braak stages I–III did not have significant [^18^F]flotaza and [^125^I]IPPI binding (Figure 6B,C), in contrast to [^125^I]α-Bgtx, where Braak stages I–III were generally higher than V–VI (Figure 6A). Each Braak stage for [^125^I]α-Bgtx exhibited a greater variability compared to those for [^18^F]flotaza and [^125^I]IPPI.

**Figure 7.**
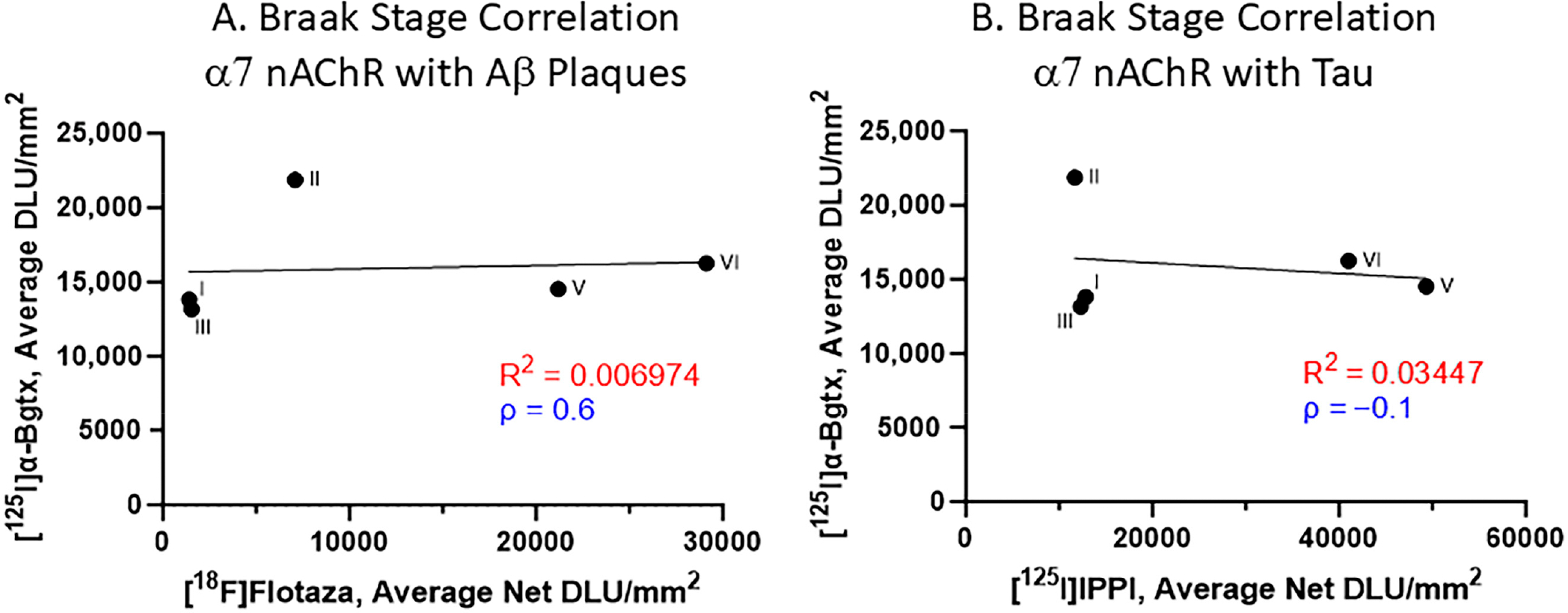
α7 nAChR binding to [^125^I]α-Bgtx correlated with Aβ plaques and Tau binding with respect to Braak stage (**A**). Braak stage Spearman’s correlation of [^125^I]α-Bgtx binding with [^18^F]flotaza binding to Aβ plaques (Spearman’s ρ = 0.6). (**B**). Braak stage Spearman’s correlation of [^125^I]α-Bgtx with [^125^I]IPPI to Tau (Spearman’s ρ = −0.1).

The impact of Aβ plaques and Tau on α7 nAChRs was evaluated with respect to the Braak staging (Figure 7). Spearman’s correlations for the relationship between [^125^I]α-Bgtx and [^18^F]flotaza indicate a positive correlation (Figure 7A). However, Spearman’s correlation revealed a negative correlation between [^125^I]α-Bgtx and [^125^I]IPPI (Figure 7B). Overall, the accumulation of Aβ plaques and Tau moderately affected [^125^I]α-Bgtx binding to α7 nAChRs in human HP.

## 4. Discussion

The HP and SUB regions play crucial roles in the cognition functions of the brain, along with cholinergic neurotransmission. This involves various pathways including α7 nAChRs that are more abundant in these brain regions. In addition, α4β2 nAChRs are also intricately involved in these brain regions [27]. During the several stages of AD, Aβ plaques and Tau affect various neurochemical targets in several brain regions. They are likely to interfere with normal cholinergic neurotransmission, as suggested by our studies on the α4β2 nAChRs [28]. Therefore, it is necessary to study the role and characteristics of α7 nAChRs in AD pathology in the HP and SUB for the translation of findings to in vivo PET studies using α7 nAChR radioligands.

The autoradiography of the postmortem brain slices allowed for easier identification of the HP and SUB regions. [^125^I]α-Bgtx was selective to the GM regions of the brain slices, specifically the HP region (Figure 2D). When compared to [^18^F]nifene, which showed higher binding to the SUB regions (Figure 2C), [^125^I]α-Bgtx demonstrated significantly more binding in the HP regions. The CA1 region of the HP and SUB have been shown to have higher levels of Aβ plaques and Tau compared to other regions of the HP in AD subjects [29]. The high expression of α7 nAChRs has been reported throughout the hippocampal circuit [30]. All of the CN subjects displayed a similar selectivity to the HP GM regions, with the male subjects showing greater variability in [^125^I]α-Bgtx binding (Figure 3). In the age correlation plots, the variability in binding was more apparent, as indicated by the lower R^2^ values (Figure 3C,F). Both the age correlation plots for the CN subjects resulted in a low negative Spearman correlation coefficient, suggesting a weak negative association. This variability may be dependent on Tau accumulation and Aβ deposition with older age despite cognitively healthy individuals [31]. The abundance of Aβ plaque and Tau was evaluated in all of the CN subjects using [^18^F]flotaza and [^125^I]IPPI, respectively. The presence of Aβ plaque and Tau in the CN subjects remained absent or significantly lower than in all of the AD subjects.

All of the male and female AD subjects were analyzed for [^125^I]α-Bgtx binding in the HP-SUB sections (Figure 4). All of the AD subjects exhibited similar binding patterns as the representative subjects, where more [^125^I]α-Bgtx binding was found in the GM compared to in the WM. Both the male and female AD subjects displayed a positive correlation between age and [^125^I]α-Bgtx binding given the positive Spearman’s correlation coefficient (Figure 4C,F). The recent finding of increased α7 nAChRs with normal aging [26] requires more careful and intricate aging studies between CN and AD subjects to elaborate on these findings. Based on previous reports, a decrease in α7 nAChRs may be expected for AD brains [32], but in our present findings, no significant difference was found between the CN and AD subjects regardless of gender (Figure 7). Despite there being no significance, [^125^I]α-Bgtx binding in the CN subjects was consistently higher than that in all of the AD subjects for both the GM and GM + WM. This discrepancy may be attributed to a possible binding complex of Aβ plaque and α7 nAChRs that induces a compensatory increase in α7 nAChR expression in response to inhibition by intensified exposure of Aβ in AD [33,34]. This increase may contribute to the higher than normal expression of α7 nAChRs found in AD and lead to less of an overall difference compared to the CN subjects; however, this relationship needs to be further investigated. The small subject size in this study may also exaggerate the variations in binding and therefore impact this discrepancy. The impact of substantial Aβ plaque pathology on α7 nAChRs implies potential connections between different AD biomarkers.

All of the CN subjects were categorized in Braak stages I–III, while all of the AD subjects were categorized in Braak stages V and VI (Figure 6). Braak stages V and VI are normally indicative of impaired cognition due to Aβ and Tau accumulation in later AD progression [10]. This accumulation in AD patients may play a role in the reduced activity of α7 nAChRs in brain regions responsible for cognition such as the HP [35]. Aβ acts as a modulator by directly affecting the function of α7 nAChRs, eventually deteriorating the presence of α7 nAChRs but activating α7 nAChRs at low Aβ concentrations. However, it is difficult to determine this direct relationship in the context of Braak staging, where a positive correlation was observed (Figure 7A). The negative correlation between the binding to α7 nAChRs and Tau implies a complementary effect of imaging multiple AD biomarkers to confirm and track AD (Figure 7B). These correlations may be associated with how α7 nAChRs mediate a high Ca^2+^ influx upon Aβ activation that may phosphorylate Tau while affecting Aβ toxicity [36]. With this, α7 nAChRs may be affected by Aβ accumulation and Tau pathology primarily in early AD. Another explanation behind this correlation may involve the neuroinflammation present in AD brains. As essential regulators of neuronal health and cholinergic signaling, α7 nAChRs play a role in managing the inflammation caused by neuritic plaques surrounded by hyper-phosphorylated Tau protein [37,38]. In response to this neuroinflammation during AD progression, there may be an upregulation of α7 nAChRs as a compensatory mechanism. Both speculations may establish a connection between Aβ and Tau pathology and α7 nAChRs. Recent studies have indicated that the efficacy of clinically used acetylcholinesterase inhibitors, AChEIs (donepezil, rivastigmine, and galantamine), in the management of AD has been less than optimal, mild, and may not be clinically significant [39,40]. The effect of AChEIs on α7 nAChRs is less clear, since the affinity of acetylcholine and nicotine is significantly weaker compared to that of α4β2 nAChRs [27]. Some benefit of AChEIs in late-onset AD (LOAD) has been observed [41]. Understanding the connection between α7 nAChRs and other AD biomarkers can provide valuable insight into effectively imaging α7 nAChRs using PET imaging agents and potentially assist in evaluating AChEIs vis-à-vis α7 nAChRs.

Limitations in this study include the small number of CN and AD subjects; the relationship between aging, sex, and α7 nAChR expression could not be more thoroughly analyzed. There have been previous reports on male/female differences in α7 RNA expression in the nucleus accumbens of AD subjects [42]. In recent PET studies of the related α4β2 nAChRs using [^18^F]nifene, no sex differences were found in several brain regions [43]. In this work, there are preliminary findings of assessing [^125^I]α-Bgtx binding to α7 nAChRs in the HP-SUB brain region that require continued studies with larger sample sizes. Because the study uses brain slices, a complete picture of the HP and SUB is difficult to acquire. Small inter-subject variations in the brain tissue may have caused some variations in the GM binding of [^125^I]α-Bgtx. Despite these limitations, this study sufficiently analyzes the potential of [^125^I]α-Bgtx as a PET imaging agent binding to α7 nAChRs in the HP-SUB regions of postmortem human AD and CN subjects. The promise of [^125^I]α-Bgtx as a supportive PET imaging agent is implied with these findings but also highlights the need to further investigate the sophisticated features of α7 nAChRs among other AD biomarkers.

## 5. Conclusions

The role of α7 nAChRs in cognitive function undoubtedly has a substantial impact on several neurological and psychiatric disorders including AD. Although this study did not find a significant difference in [^125^I]α-Bgtx binding between the AD and CN subjects, [^125^I]α-Bgtx is still useful as a complementary tool in the study of α7 nAChRs [44]. The findings of our study appear to be consistent with the reported findings of the characteristics of α7 nAChRs in the HP-SUB region; however, regarding the discrepancies in α7 nAChR expression, future studies should aim to comprehensively understand the nature of α7 nAChR expression in AD. Transgenic AD mice studies expressing Aβ plaques in 5xFAD mice and Aβ plaques and tau in 3xTg mice [45] may be useful in longitudinal PET studies to study alterations in α7 nAChRs in transgenic mice models [46,47].

## Figures and Tables

**Table 1.** Patient samples and data *.

Subjects, N	CERAD Pathology	Gender	Age Range, Mean ± SD	PMI, hrs	Brain Region	Plaque Total	Tangle Total	LB	Braak Score
13	CN	Male	71–97 (79.9 ± 8.55)	2–5.4	HP	0–5.5	0–6	0	I–III
15	CN	Female	53–95 (80.4 ± 13.1)	2.1–4.8	HP	0–10	0.5–6.5	0	I–III
13	AD	Male	70–91 (80.4 ± 5.98)	2.3–4.8	HP	14–15	10–15	0	V–VI
16	AD	Female	59–93 (81.3 ± 9.26)	1.8–5	HP	10–15	12–15	0	V–VI

* Frozen brain samples were obtained from Banner Sun Health Institute, Sun City, Arizona [24]

CN = cognitively normal and may include mild cognitive impairment (MCI) subjects; AD = Alzheimer’s disease; PMI = postmortem interval in hours; HP = hippocampus; LB = Lewy Bodies. Plaque total includes neuritic, cored, and diffuse, in frontal, temporal, parietal, hippocampal, and entorhinal cortex. Semi-quantitative scores of none, sparse, moderate, and frequent were converted into numerical values 0–3 for each region and summed to provide the plaque total. Tangle total: Neurofibrillary tangle density in frontal, temporal, and parietal lobes, hippocampal CA1 region and entorhinal cortical regions. Numerical values 0–3 for each region were summed to provide tangle total. Braak score: Braak neurofibrillary stage (0–VI). Brain slices (10 μm thickness) were obtained from the chunks of frozen tissue on a Leica 1850 cryotome (LeicaMicrosystems, Inc., Deer Park, IL, USA) cooled to −20 °C and collected on Fisher slides.

## Data Availability

The data that support the findings of this study are available from the corresponding author upon discussion after reasonable request. In addition, the data will be uploaded to University of California, Irvine’s managed website, https://faculty.sites.uci.edu/mukherjeelabs/data-sharing/, and can be accessed anonymously.
